# Racial and Ethnic Subgroup Disparities in Hypertension Prevalence, New York City Health and Nutrition Examination Survey, 2013–2014

**DOI:** 10.5888/pcd14.160478

**Published:** 2017-04-20

**Authors:** Kezhen Fei, Jesica S. Rodriguez-Lopez, Marcel Ramos, Nadia Islam, Chau Trinh-Shevrin, Stella S. Yi, Claudia Chernov, Sharon E. Perlman, Lorna E. Thorpe

**Affiliations:** 1 Graduate School of Public Health and Health Sciences, City University of New York, New York, New York; 2 Department of Population Health and Science, Icahn School of Medicine at Mount Sinai, New York, New York; 3 Departamento de Ingeniería Industrial, Universidad de La Salle, Bogotá, Colombia; 4 Department of Population Health, New York University School of Medicine, New York, New York; 5 Division of Epidemiology, New York City Department of Health and Mental Hygiene, Queens, New York

## Abstract

**Introduction:**

Racial/ethnic minority adults have higher rates of hypertension than non-Hispanic white adults. We examined the prevalence of hypertension among Hispanic and Asian subgroups in New York City.

**Methods:**

Data from the 2013–2014 New York City Health and Nutrition Examination Survey were used to assess hypertension prevalence among adults (aged ≥20) in New York City (n = 1,476). Hypertension was measured (systolic blood pressure ≥140 mm Hg or diastolic blood pressure ≥90 mm Hg or self-reported hypertension and use of blood pressure medication). Participants self-reported race/ethnicity and country of origin. Multivariable logistic regression models assessed differences in prevalence by race/ethnicity and sociodemographic and health-related characteristics.

**Results:**

Overall hypertension prevalence among adults in New York City was 33.9% (43.5% for non-Hispanic blacks, 38.0% for Asians, 33.0% for Hispanics, and 27.5% for non-Hispanic whites). Among Hispanic adults, prevalence was 39.4% for Dominican, 34.2% for Puerto Rican, and 27.5% for Central/South American adults. Among Asian adults, prevalence was 43.0% for South Asian and 39.9% for East/Southeast Asian adults. Adjusting for age, sex, education, and body mass index, 2 major racial/ethnic minority groups had higher odds of hypertension than non-Hispanic whites: non-Hispanic black (AOR [adjusted odds ratio], 2.6; 95% confidence interval [CI], 1.7–3.9) and Asian (AOR, 2.0; 95% CI, 1.2–3.4) adults. Two subgroups had greater odds of hypertension than the non-Hispanic white group: East/Southeast Asian adults (AOR, 2.8; 95% CI, 1.6–4.9) and Dominican adults (AOR, 1.9; 95% CI, 1.1–3.5).

**Conclusion:**

Racial/ethnic minority subgroups vary in hypertension prevalence, suggesting the need for targeted interventions.

**MEDSCAPE CME**

Medscape, LLC is pleased to provide online continuing medical education (CME) for this journal article, allowing clinicians the opportunity to earn CME credit.

This activity has been planned and implemented through the joint providership of Medscape, LLC and *Preventing Chronic Disease*. Medscape, LLC is accredited by the American Nurses Credentialing Center (ANCC), the Accreditation Council for Pharmacy Education (ACPE), and the Accreditation Council for Continuing Medical Education (ACCME), to provide continuing education for the healthcare team.

Medscape, LLC designates this Journal-based CME activity for a maximum of 1.00 *AMA PRA Category 1 Credit(s)™*. Physicians should claim only the credit commensurate with the extent of their participation in the activity.

All other clinicians completing this activity will be issued a certificate of participation. To participate in this journal CME activity: (1) review the learning objectives and author disclosures; (2) study the education content; (3) take the post-test with a 75% minimum passing score and complete the evaluation at http://www.medscape.org/journal/pcd; (4) view/print certificate.

**Release date: April 20, 2017; Expiration date: April 20, 2018**

**Learning Objectives**

Upon completion of this activity, participants will be able to:

1. Distinguish the overall prevalence of hypertension in the current study sample
2. Assess which racial/ethnic group has the highest age-standardized prevalence of hypertension in the current study
3. Evaluate differences in the adjusted odds for hypertension based on race/ethnicity in adults
4. Analyze the effect of body mass index on the prevalence of hypertension among different racial/ethnic groups

**EDITOR**

Ellen Taratus, MS, ELS

Editor, *Preventing Chronic Disease*

Disclosure: Ellen Taratus has disclosed no relevant financial relationships.

**CME AUTHOR**

Charles P. Vega, MD

Health Sciences Clinical Professor, UC Irvine Department of Family Medicine; Associate Dean for Diversity and Inclusion, UC Irvine School of Medicine, Irvine, California

Disclosure: Charles P. Vega, MD, has disclosed the following relevant financial relationships:

Served as an advisor or consultant for: McNeil Consumer Healthcare

Served as a speaker or a member of a speakers bureau for: Shire Pharmaceuticals

**AUTHORS**

Kezhen Fei, MS

Graduate School of Public Health and Health Sciences, City University of New York; Department of Population Health and Science, Icahn School of Medicine at Mount Sinai, New York, New York

Disclosure: Kezhen Fei, MS, has disclosed no relevant financial relationships.

Jesica S. Rodriguez-Lopez, MPH

Graduate School of Public Health and Health Sciences, City University of New York, New York, New York

Disclosure: Jesica S. Rodriguez-Lopez, MPH, has disclosed no relevant financial relationships.

Marcel Ramos, MPH

Graduate School of Public Health and Health Sciences, City University of New York, New York, New York

Disclosure: Marcel Ramos, MPH, has disclosed no relevant financial relationships.

Nadia Islam, PhD

Department of Population Health, New York University School of Medicine, New York, New York

Disclosure: Nadia Islam, PhD, has disclosed no relevant financial relationships.

Chau Trinh-Shevrin, DrPH

Department of Population Health, New York University School of Medicine, New York, New York

Disclosure: Chau Trinh-Shevrin, DrPH, has disclosed no relevant financial relationships.

Stella S. Yi, PhD

Department of Population Health, New York University School of Medicine, New York, New York

Disclosure: Stella S. Yi, PhD, has disclosed no relevant financial relationships.

Claudia Chernov, MPH

Division of Epidemiology, New York City Department of Health and Mental Hygiene, Queens, New York

Disclosure: Claudia Chernov, MPH, has disclosed no relevant financial relationships.

Sharon E. Perlman, MPH

Division of Epidemiology, New York City Department of Health and Mental Hygiene, Queens, New York

Disclosure: Sharon E. Perlman, MPH, has disclosed no relevant financial relationships.

Lorna E. Thorpe, PhD

Department of Population Health, New York University School of Medicine, New York, New York

Disclosure: Lorna E. Thorpe, PhD, has disclosed no relevant financial relationships.

## Introduction

Hypertension is a major risk factor for cardiovascular disease and worsens outcomes for people with diabetes or kidney disease (1–4). The 1960s Charleston Heart Study and other cohort studies show higher prevalence of hypertension among black participants than among white participants (5,6). More recently, National Health and Nutrition Examination Survey (NHANES) data from 1999–2010 showed a higher prevalence of hypertension among black adults than among white or Mexican American adults (black men [39.6%], white men [29.8%], Mexican American men [26.4%], black women [43.1%], white women [26.9%], Mexican American women [27.7%]), with stable rates of disparities from 1999 to 2010 (7). In 2011–2014, NHANES oversampled Asian and Hispanic participants to produce reliable estimates; hypertension prevalence among non-Hispanic Asian adults (24.9%) and Hispanic adults (25.9%) was similar and lower than the prevalence among non-Hispanic white adults (28.0%) (8). To our knowledge, few population-based studies have examined differences among Hispanic and Asian subgroups.

Recent health examination data collected from racially/ethnically diverse urban settings could shed light on the heterogeneity of data on hypertension prevalence among racial/ethnic subgroups. For example, the Hispanic Community Health Study/Study of Latinos is a longitudinal cohort of 16,415 urban Hispanic adults in the United States. Although the study is not population-based, it estimated the prevalence of hypertension at its Bronx site as 29.5% among Dominicans, 28.6% among Puerto Ricans, and 26.6% among Central Americans, and a significantly lower prevalence of 13.3% among Mexican Americans (9). The Multi-Ethnic Study of Atherosclerosis also found lower hypertension prevalence among Mexican Americans than among other Hispanic subgroups (10).

In 2004, the New York City Health and Nutrition Examination Survey (NYC HANES), modeled after NHANES, measured blood pressure in a population-based sample of adults in New York City aged 20 or older (11). NYC HANES 2004 was the first population-based study to examine differences in hypertension prevalence among Asian and Hispanic subgroups. Following NHANES measurement protocols, researchers measured the blood pressure of participants in clinics using a mercury manometer and estimated an hypertension prevalence of 25.5% among adults in New York City overall, 32.8% among black adults, 26.4% among Hispanic adults, 24.7% among Asian adults, and 21.1% among non-Hispanic white adults.

The objective of our study was to describe the prevalence of hypertension among adults in major racial/ethnic minority population groups and among Asian and Hispanic subgroups using data from NYC HANES 2013–2014 before and after adjusting for demographic characteristics. Because of the rapidly changing composition of the population in New York City, monitoring the prevalence of hypertension by racial/ethnic categories is important. We hypothesized that the prevalence of hypertension among adults in Hispanic and Asian subgroups would differ from the prevalence among non-Hispanic white adults.

## Methods

NYC HANES is a population-based, cross-sectional survey of adults in New York City. Data for the most recent survey were collected from August 2013 through June 2014; details of the study design are available elsewhere (12). Briefly, a probability-based, 3-stage clustering design was used to select households in New York City. The survey included 3 components: an in-person interview, a physical examination (to measure blood pressure, pulse, height, weight, and waist circumference), and biological specimen collection. All participants gave informed consent. The survey was conducted in English, Spanish, Russian, Mandarin, or Cantonese, with telephone translation available for other non–English-speaking participants. The study protocol was approved by the institutional review boards of the City University of New York School of Public Health, the New York City Department of Health and Mental Hygiene, and RTI International. The overall response rate was 36%; 1,527 individuals completed the survey. Differences between unweighted and weighted demographic distributions were modest and nonsignificant, suggesting that the final sample was broadly representative of the city’s population (12). 

For this analysis, we included all participants in NYC HANES who were not pregnant and had either valid blood pressure measurements or information on hypertension diagnosis or medication. Twenty pregnant women were excluded, and 31 participants were excluded because of either invalid blood pressure measurements or missing information on hypertension diagnosis or medication; on average, these 31 participants did not differ from the final sample on age, sex, race/ethnicity, body mass index (BMI), or education. The final analytic sample consisted of 1,476 adults. Before the study, we calculated that the sample size required to estimate the prevalence of a condition with a prevalence range similar to that of hypertension (25%–30%) with a margin of error of ±4.0% was 1,800 to 1,935 participants.

To compare NHANES 2013–2014 data on hypertension prevalence with national data, we downloaded national data from the Centers for Disease Control and Prevention and examined differences by sex, income, and education (13).

### Measures

The instrument used to measure blood pressure in the 2013–2014 NYC HANES differed from that used in 2004. Instead of a mercury sphygmomanometer (11), an automatic inflatable digital blood pressure monitor with 4 cuff sizes (LifeSource UA-789AC, A&D Medical Ltd) was used to measure blood pressure in the participant’s home (12); 3 measurements were taken for each participant. The mean of the second and third values was used as the final measurement. Blood pressure measurements determined by this device were validated as equivalent by the American National Standards Institute to measurements determined by an electronic sphygmomanometer (14). Hypertension was defined as systolic blood pressure of 140 mm Hg or more, diastolic blood pressure of 90 mm Hg or more, or self-reported hypertension diagnosis and current use of prescribed antihypertensive medication (15). Weight was measured to the nearest 0.1 kg and height to the nearest 0.5 cm. BMI was calculated as weight in kilograms divided by height in meters squared (kg/m^2^); BMI categories were classified according to NHANES protocol (16). BMI in our sample ranged from 13 to 69. Heavy alcohol use was defined as more than 2 drinks per day and every day for men, and more than 1 drink per day and every day for women. One drink was explained to participants as a 12-ounce beer, a 5-ounce glass of wine, or one-and-a-half ounces of liquor. Current smoker was defined as someone who answered yes to “Have you smoked at least 100 cigarettes in your entire life” and stated that he or she currently smokes some days or every day.

Categorization of a participant’s major racial/ethnic group was based on the participant’s responses to the following questions, which are used in NHANES (16): “Do you consider yourself as Hispanic/Latino?” and “What race/races do you consider yourself?” Adults were categorized into 5 mutually exclusive major race/ethnicity groups: non-Hispanic white (white), non-Hispanic black (black), non-Hispanic Asian (Asian), Hispanic, and non-Hispanic other. Seventy “non-Hispanic other” adults were excluded from group analysis because of small sample size. Asian adults were further categorized as East/Southeast Asian or as South Asian according to responses to questions about their Asian origin and ancestry. East/Southeast Asian adults included those of Chinese, Japanese, Korean, Filipino, Laos, Thai, Cambodian, and Vietnamese origin. South Asian adults included those of Bangladeshi, Indian, East Indian, Asian Indian, Nepalese, Pakistani, Sri Lankan, and Goan origin. Hispanics were further categorized as Puerto Rican, Dominican, or Central/South American based on responses to questions about their Hispanic/Latino origin or ancestry. Central/South American adults included those of Mexican, Cuban, Costa Rican, Guatemalan, Honduran, Nicaraguan, Panamanian, Salvadoran, Argentinean, Bolivian, Chilean, Colombian, Ecuadorian, Paraguayan, Peruvian, Uruguayan, Venezuelan, and other Central and South American origin or ancestry.

### Statistical analyses

Statistical analyses were weighted to adjust for the complex sampling design, nonresponse, and poststratification. A design poststratification weight was created to represent the New York City population by age, sex, race/ethnicity, borough of residence, education, and marital status, using the American Community Survey 2013 (17). Weights were then further adjusted for item-level nonresponse (12). SAS version 9.4 (SAS Institute, Inc) was used to perform all analyses. Prevalence estimates were age standardized to the 2000 US population (18). Relative standard errors were calculated for each estimate to assess reliability; none, however, were above 30%. Rao–Scott χ^2 ^tests were used for bivariate comparisons. Multivariable logistic regression was used to assess racial/ethnic differences by adjusting for age, sex, education, and BMI. We did not estimate changes in hypertension prevalence between the 2004 NYC HANES and the 2013–2014 NYC HANES because each survey used a different method for measuring blood pressure.

Effect modification between race and sex, education, and BMI on hypertension was assessed by adding individual interaction terms in multivariable logistic regression; we performed further stratified analysis only if a significant interaction was found. Statistical significance level was set at .05.

## Results

The racial/ethnic distribution of NYC HANES 2013–14 was diverse: 35.0% were white, 27.1% were Hispanic, 21.3% were black, and 14.2% were Asian. Asian participants were younger than those in other major racial/ethnic groups (*P* = .01) (Table 1). A greater proportion of Hispanic adults than adults in other major racial/ethnic groups had less than a high school education and less than $20,000 in annual household income (*P* < .001). We found a higher proportion of women among black adults than that among white adults (*P* = .03). A greater proportion of white adults than adults in other major racial/ethnic groups had private health insurance coverage (*P* < .001). Black and Hispanic adults had a greater prevalence of obesity than did white or Asian adults (black, 36.9% and Hispanic, 36.8% vs white, 27.6% and Asian, 14.9%; *P* < .001). The prevalence of smoking did not significantly differ across major racial/ethnic groups.

**Table 1:** Demographic and Behavioral Characteristics by Racial/Ethnic Groups and Subgroups Among Adults in New York City, New York City Health and Nutrition Examination Survey, 2013–2014^a^

Characteristic	All Adults	Major Racial/Ethnic Group^b^				Hispanic Subgroup^b^			Non-Hispanic Asian Subgroup^b^	
		Non-Hispanic White	Non-Hispanic Black	Hispanic	Non-Hispanic Asian	Puerto Rican	Dominican	Central or South American^c^	East or Southeast Asian^d^	South Asian^e^
**Total^b^**	1,476 (100.0)	495 (35.0)	328 (21.3)	382 (27.1)	200 (14.2)	143 (36.0)	92 (23.1)	137 (38.4)	131 (62.4)	60 (31.8)
**Age group, y^f^**										
<50	910 (59.9)	287 (54.8)	202 (61.2)	234 (60.2)	145 (70.9)	84 (58.6)	50 (53.1)	92 (65.0)	93 (68.7)	47 (80.6)
≥50	566 (40.1)	208 (45.2)	126 (38.8)	148 (39.8)	55 (29.1)	59 (41.4)	42 (46.9)	45 (35.0)	38 (31.3)	13 (20.3)
**Sex^f^^,^ g**										
Male	632 (46.8)	237 (51.4)	122 (41.0)	156 (45.1)	84 (46.4)	61 (46.5)	28 (32.2)	60 (49.4)	57 (47.8)	24 (43.6)
Female	844 (53.2)	258 (48.6)	206 (59.0)	226 (54.9)	116 (53.6)	82 (53.5)	64 (67.8)	77 (50.6)	74 (52.2)	36 (56.4)
**Education^f^^,^ g ^,^ h**										
<High school diploma	307 (18.7)	28 (5.8)	83 (20.0)	149 (35.1)	36 (17.0)	63 (39.7)	44 (46.8)	38 (24.3)	18 (14.0)	17 (25.0)
High school diploma	235 (23.6)	48 (14.7)	78 (33.6)	78 (30.3)	25 (20.3)	27 (28.8)	12 (19.0)	37 (38.2)	12 (14.4)	9 (22.9)
>High school diploma	932 (57.7)	418 (79.4)	167 (46.4)	154 (34.6)	139 (62.7)	53 (31.5)	35 (34.1)	62 (37.5)	101 (71.6)	34 (52.2)
**Annual household income^f^^,^ g**										
<$20,000	399 (28.6)	77 (15.6)	97 (31.7)	155 (44.2)	52 (29.5)	57 (46.6)	49 (56.8)	45 (35.0)	31 (26.5)	19 (36.9)
≥$20,000	986 (71.4)	405 (84.4)	199 (68.3)	193 (55.8)	138 (70.5)	68 (53.4)	37 (43.2)	82 (65.0)	96 (73.5)	31 (63.1)
**Health insurance coverage^f^^,^ g**										
Private	649 (43.1)	285 (56.5)	132 (39.7)	117 (30.6)	85 (39.4)	48 (33.7)	19 (20.9)	47 (33.5)	57 (39.3)	25 (42.9)
Medicare/government	565 (39.4)	142 (30.9)	134 (42.6)	186 (48.8)	73 (37.3)	74 (52.0)	59 (65.0)	48 (35.5)	46 (36.5)	24 (40.4)
Uninsured	248 (17.4)	66 (12.7)	55 (17.7)	76 (20.7)	41 (23.3)	19 (14.4)	14 (14.1)	41 (31.0)	28 (24.1)	10 (16.6)
**Health behavior**										
Current smoker^g^^,^ i	277 (18.9)	86 (17.7)	73 (23.1)	70 (17.4)	35 (17.9)	48 (32.9)	6 (7.5)	14 (8.8)	25 (18.3)	8 (16.0)
Heavy alcohol use^j^	107 (6.7)	42 (6.8)	21 (6.1)	36 (9.2)	6 (3.1)	12 (7.9)	6 (6.3)	17 (12.3)	4 (4.1)	2 (1.6)
BMI >30.0^f^^,^ h	424 (30.4)	121 (27.6)	121 (36.9)	134 (36.8)	27 (14.9)	52 (38.9)	35 (43.5)	45 (32.3)	13 (9.9)	11 (18.4)

Abbreviation: BMI, body mass index.

a Data are unweighted n (weighted %). Weights were to adjust for the complex sampling design, nonresponse, and poststratification. A design weight equal to the inverse of the probability of household selection was applied to each household. A household-level nonresponse adjustment factor was then applied, and final weighting involved raking sample weights, so adjusted weights added to known marginal population totals for poststratification categories of age, sex, race/ethnicity, borough, education and marital status, per the 2013 American Community Survey (17), to represent the New York City population.

b Numbers do not total to 100% because estimates are not shown for non-Hispanic “other” (n = 71) in major racial/ethnic categories, for Hispanic “other” (n = 10) in Hispanic subgroups, or for Asian “other” (n = 9) in Asian subgroup.

c Central/South American includes Mexican, Cuban, Costa Rican, Guatemalan, Honduran, Nicaraguan, Panamanian, Salvadoran, Argentinean, Bolivian, Chilean, Colombian, Ecuadorian, Paraguayan, Peruvian, Uruguayan, Venezuelan, and other Central and South American.

d East/Southeast Asian includes Chinese, Japanese, Korean, Filipino, Laotian, Thai, Cambodian, and Vietnamese.

e South Asian includes Bangladeshi, Indian, East Indian, Asian Indian, Nepalese, Pakistani, Sri Lankan, and Goan.

f *P* < .05 across all major racial/ethnic groups.

g *P* < .05 across all Hispanic subgroups.

h *P* < .05 across all Asian subgroups.

i Current smoker was defined as someone who answered yes to “Have you smoked at least 100 cigarettes in your entire life” and stated that he or she currently smokes some days or every day.

j Men who indicated having >2 drinks per day every day and women who indicated having >1 drink per day every day.

Within Hispanic and Asian subgroups, demographic profiles and health behaviors varied. Among Hispanic adults, the largest subgroup was from Central and South America (38.4%), followed by Puerto Rico (36.0%), and the Dominican Republic (23.1%). Among Asian adults, 62.4% were of East/Southeast Asian origin, and 31.8% were of South Asian origin. Among Hispanic subgroups, adults from the Dominican Republic had the greatest proportion of women (*P* = .004) and the greatest percentage of adults with less than a high school education (*P* = .05). Compared with other Hispanic subgroups, a greater proportion of Dominicans had Medicaid/Medicare or other government health insurance and a lower proportion had private health insurance. Central/South Americans had the greatest proportion of uninsured adults (*P* = .002). A greater proportion of Puerto Rican adults were current smokers compared with Dominican and Central/South American adults (Puerto Rican, 32.9% vs Dominican, 7.5%, and Central/South American, 8.8%; *P* < .001). Among Asians, East/Southeast Asian adults had a greater proportion of adults with more than a high school education than South Asians (71.6% vs 52.2%, *P* < .001). A greater proportion of South Asian adults were obese compared with East/Southeast Asian adults (18.4% vs 9.9%, *P* = .001). 

The overall prevalence of hypertension among adults in New York City was 33.9% and increased with age (Table 2). Prevalence was 10.4% among adults aged 20 to 39, 40.2% among those aged 40 to 59, and 64.0% among those aged 60 or older. After age standardization, men were slightly more likely than women to have hypertension (36.2% vs 31.8%, *P* = .01). White adults had a significantly lower rate of hypertension than black, Asian, or Hispanic adults: the age-standardized prevalence was 27.5% for white, 43.5% for black, 38.0% for Asian, and 33.0% for Hispanic adults. Age-standardized hypertension prevalence was significantly higher among adults from South Asia (43.0%), East/Southeast Asia (39.9%), and the Dominican Republic (39.4%) than among white adults (27.5%) (*P* < .001).

**Table 2:** Hypertension^a^ Prevalence by Age, Sex, Race/Ethnicity, Education, and Body Mass Index Among Adults in New York City, New York City Health and Nutrition Examination Survey, 2013–2014

Characteristic	Unweighted Total^b^	Weighted Total	Weighted and Age Standardized %^c^ (95% Confidence Interval)	*P *Value^d^
**Overall**	1,476	6,285,749	33.9 (31.4−36.4)	—
**Sex**				
Male	632	2,942,712	36.2 (32.5−40.0)	.01
Female	844	3,343,037	31.8 (28.5−35.1)	
**Age group, y**				
20–39	668	2,630,758	10.4 (7.7−13.1)	<.001
40–59	499	2,194,045	40.2 (35.4−44.9)	
≥60	309	1,460,946	64.0 (58.0−69.9)	
**Race/ethnicity**				
Non-Hispanic white	495	2,201,667	27.5 (23.5−31.4)	<.001
Non-Hispanic black	328	1,336,586	43.5 (38.2−48.8)	
Hispanic	382	1,703964	33.0 (28.4−37.7)	
Non-Hispanic Asian	200	889,666	38.0 (30.4−45.6)	
**Racial/ethnic subgroup**				
Non-Hispanic white	495	2,201,667	27.5 (23.5−31.4)	<.001
Non-Hispanic black	328	1,336,586	43.5 (38.2−48.8)	
Puerto Rican	143	613,036	34.2 (26.3−42.2)	
Dominican	92	394,423	39.4 (29.9−49.0)	
Central or South American	137	654,180	27.5 (19.8−35.3)	
East or Southeast Asian	131	554,993	39.9 (31.0−48.7)	
South Asian	60	282,881	43.0 (32.1−53.8)	
**Education**				
<High school diploma	307	1,171,585	38.3 (33.8−42.9)	.01
High school diploma	235	1,484,645	35.0 (28.9−41.1)	
>High school diploma	932	3,622,619	31.0 (27.8−34.2)	
**Body mass index**				
Normal/underweight	545	2,185,414	23.6 (19.5−27.7)	<.001
Overweight	486	2,106,612	30.3 (26.3−34.4)	
Obese	424	1,874,573	46.5 (41.8−51.3)	

a Hypertension is defined as currently taking antihypertensive medication or having systolic blood pressure ≥140 mm Hg or diastolic blood pressure ≥90 mm Hg.

b Categories may not add to 1,476 because not all participants answered questions on racial/ethnic subgroup or education and the records of 21 participants lacked information on body mass index.

c Relative standard error for all estimates was <30%; the largest was 17%.

d *P* value determined by Rao–Scott χ^2^ test, which compared within group difference on age-adjusted hypertension prevalence.

In multivariate logistic regression, after adjusting for age, sex, education, and BMI, black and Asian adults had significantly greater odds of hypertension than whites (black, adjusted odds ratio [AOR], 2.6; 95% CI, 1.7–3.9; Asian, AOR, 2.0; 95% CI, 1.2–3.4), but adjusted odds for Hispanic and white adults were similar (Table 3). After adjustment, Puerto Rican, Central/South American, and South Asian adults had odds of hypertension similar to those for whites, but Dominican adults had nearly twice the odds of white adults (AOR, 1.9; 95% CI, 1.1–3.5). East/Southeast Asian adults had the greatest odds of hypertension, nearly 3 times that of white adults (AOR, 2.8; 95% CI, 1.6–4.9).

**Table 3:** Logistic Regression With Race as a Predictor for Hypertension Among Adults in New York City, New York City Health and Nutrition Examination Survey, 2013–2014

Racial/Ethnic Group	No.	OR (95% CI)				
		Age Adjusted	Adjusted for Age, Sex, Education, and BMI	Adjusted for Age, Sex and Education and Stratified by BMI Group		
				Normal or Underweight	Overweight	Obese
**By major group**						
Non-Hispanic white	495	1.0 [Ref]	1.0 [Ref]	1.0 [Ref]	1.0 [Ref]	1.0 [Ref]
Non-Hispanic black	328	2.5 (1.7−3.7)	2.6 (1.7−3.9)	6.6 (2.7−16.0)	2.6 (1.4−4.9)	1.6 (0.8−3.1)
Hispanic	382	1.4 (0.9−2.1)	1.3 (0.9−2.0)	3.5 (1.4−8.7)	1.4 (0.7−2.5)	0.8 (0.4−1.5)
Non-Hispanic Asian	200	1.7 (1.1−2.9)	2.0 (1.2−3.4)	5.8 (2.3−14.9)	2.6 (1.2−5.5)	0.5 (0.2−1.6)
**By major group and subgroup^a^**						
Non-Hispanic white	495	1.0 [Ref]	1.0 [Ref]	1.0 [Ref]	1.0 [Ref]	1.0 [Ref]
Non-Hispanic black	328	2.5 (1.7−3.7)	2.5 (1.7−3.8)	6.8 (2.8−16.6)	2.6 (1.3−4.8)	1.5 (0.8−3.1)
Puerto Rican	143	1.5 (0.9−2.5)	1.4 (0.8−2.3)	3.6 (1.0−12.4)	1.5 (0.7−3.3)	0.8 (0.3−1.7)
Dominican	92	2.1 (1.2−3.8)	1.9 (1.1−3.5)	3.4 (0.8−14.0)	1.1 (0.5−2.5)	2.1 (0.8−5.4)
Central or South American	137	1.0 (0.6−1.7)	1.0 (0.5−1.7)	4.5 (1.4−14.3)	1.3 (0.5−2.9)	0.4 (0.2−0.9)
East or Southeast Asian	131	1.9 (1.1−3.3)	2.8 (1.6−4.9)	7.0 (2.5−19.3)	2.7 (1.2−6.4)	1.8 (0.5−6.2)
South Asian	60	1.7 (0.7−4.1)	1.5 (0.7−3.2)	3.6 (1.1−11.8)	2.2 (0.7−6.7)	0.4 (0.1−1.9)

Abbreviations: BMI, body mass index; CI, confidence interval; OR, odds ratio; Ref, reference.

a Not all participants in Hispanic or Asian categories answered question on racial/ethnic subgroup.

A significant interaction (*P* = .002) between race and BMI indicated a potential differential effect of BMI on hypertension across racial/ethnic groups. After stratifying analyses by BMI group, we found that prevalence of hypertension increased monotonically as BMI increased among white, black, and Hispanic adults but not among Asian adults (Figure). In the normal/underweight category (Table 3), hypertension prevalence among black, Hispanic, and Asian adults differed significantly from prevalence among white adults. Among normal/underweight people, non-Hispanic black (AOR, 6.6; 95% CI, 2.7–16.0) and Asian adults (AOR, 5.8; 95% CI, 2.3–14.9) had approximately 6 times greater odds of hypertension than white adults, whereas Hispanics had 3.5 (95% CI, 1.4–8.7) times greater odds of hypertension than white adults. Normal/underweight East/Southeast Asian adults had the greatest odds of hypertension (AOR, 7.0; 95% CI, 2.5–19.3) compared with normal/underweight white adults. Normal/underweight Central/South Americans had 4.5 times greater odds (95% CI, 1.4–14.3), and Puerto Ricans and South Asians had 3.6 times greater odds (95% CI, 1.0–12.4 for Puerto Ricans; 1.1–11.8 for South Asians) of hypertension than normal/underweight white adults. Among overweight adults, only black adults (AOR, 2.6; 95% CI, 1.3–4.8) and East/Southeast Asian adults (AOR, 2.7; 95% CI, 1.2–6.4) had a significantly higher prevalence of hypertension than white adults. Among obese adults, we found no differences in hypertension between white adults and adults in the other 3 major racial/ethnic groups; among subgroups, only obese Central/South American adults had lower odds of hypertension than obese white adults (AOR = 0.4; 95% CI, 0.2–0.9).

**Figure:**
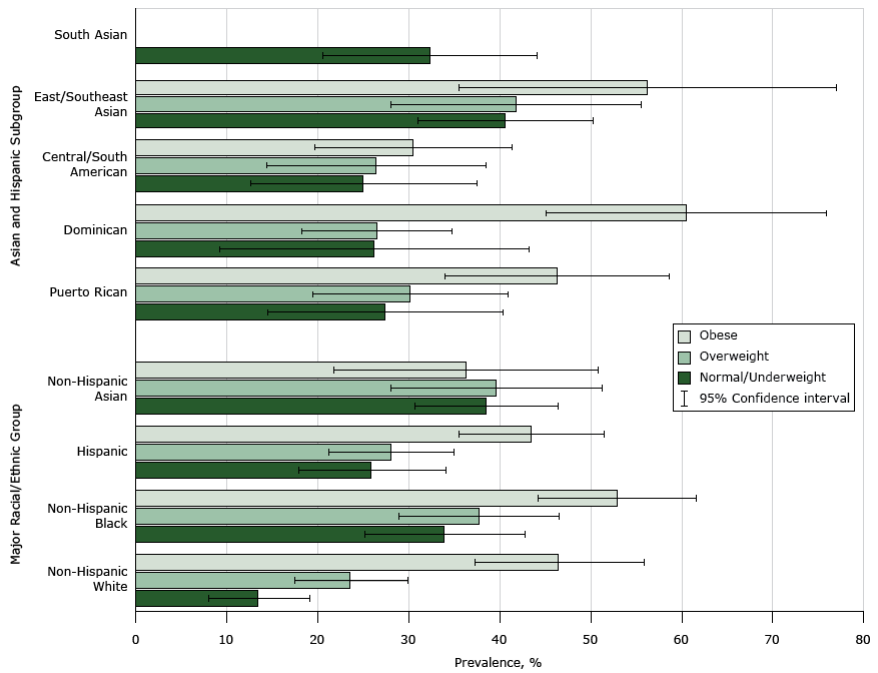
Prevalence of age standardized hypertension by major racial/ethnic group, Hispanic and Asian subgroups, and body mass index, New York City Health and Nutrition Examination Survey, 2013–2014. Relative standard errors for estimates were <30% for all races and ethnicities, except normal/underweight Dominicans (33%). We could not produce reliable estimates for South Asians in the overweight and obese categories, so no bars appear for those categories.

## Discussion

We estimated hypertension prevalence for racial/ethnic groups using a population-based sample of adults in an ethnically/racially diverse urban setting. In addition to confirming a greater prevalence of hypertension among black adults, we found substantial differences among racial/ethnic groups, even after adjusting for BMI, age, and sociodemographic characteristics. In particular, we observed significantly greater hypertension prevalence among Asian adults than among white adults. We also found that, once subgroup differences in age, education, gender and BMI were taken into account, larger proportions of adults from East/Southeast Asia and from Dominican Republic had hypertension, and differences in hypertension prevalence among racial/ethnic subgroups was especially pronounced among normal/underweight adults.

Our study found greater prevalence of hypertension among Hispanic adults in New York City than Yoon et al found in a national sample of Hispanic adults (33.0% vs 25.9%) (8). Hispanic New Yorkers differ from Hispanic Americans elsewhere both in their country of origin and in income. Whereas 44.2% of Hispanic adults participating in NYC HANES had an annual household income less than $20,000 in 2013–2014, only 26.8% of Hispanics participating in NHANES had annual household income less than $20,000 in 2013–2014. Low socioeconomic status is associated with a greater risk of hypertension (19,20). Moreover, the Hispanic Community Health Study/Study of Latinos showed that age-adjusted hypertension prevalence among Hispanic subgroups varied significantly among cities. For example, Central/South Americans in Chicago had significantly lower prevalence of hypertension than Central/South Americans in the Bronx or Miami (9).

Our study found that Dominican adults had a significantly higher prevalence of hypertension than white adults, consistent with other community- and population-based studies showing greater prevalence of hypertension among Dominicans than among whites (9,21,22). We also found hypertension prevalence to be high among Puerto Rican adults, but the disparity between Puerto Rican adults and white adults was not as marked as the disparity between Dominican adults and white adults, especially after adjusting for BMI.

Our study found significantly greater prevalence of hypertension among Asian adults in New York City than Yoon et al found among Asian adults in a national sample (38.0% vs 24.9%) (8). NYC HANES estimates of hypertension prevalence among Asian subgroups, however, were similar to estimates in the Multi-Ethnic Study of Atherosclerosis and the Mediators of Atherosclerosis in South Asians Living in America study (MASALA), community-based cohort studies carried out in the San Francisco Bay area and around Chicago. Hypertension prevalence among Chinese adults in our study was 35.6%, compared with 39% in the Multi-Ethnic Study of Atherosclerosis, and our hypertension estimate for South Asian adults was 43.0%, compared with 41% in MASALA (23,24). Higher hypertension prevalence among Asian adults in New York City than among Asian adults nationally may be explained by differences in country of origin or in socioeconomic characteristics (25). Our study found that 29.5% of Asian adults had annual household income less than $20,000 and 37.3% had only a high school diploma or less. In contrast, only 12% of Asians participating in NHANES had annual household income less than $20,000 and only 27% had only a high school diploma or less. 

Asian adults in New York City had a significantly higher prevalence of hypertension than white adults. This elevated prevalence corresponds with elevated mortality from hypertensive heart disease and cerebrovascular disease, especially hemorrhagic stroke, among Asian Americans compared with white Americans (26). Unadjusted hypertension prevalence was particularly high among South Asian adults in our study, but when we accounted for age, education, and obesity, the prevalence of hypertension was highest among East/Southeast Asian adults. The odds of hypertension among nonoverweight Asian adults was greater than among nonoverweight white adults, suggesting that Asians are more vulnerable to hypertension at lower BMI, similar to the phenomenon observed with diabetes (27). Two other studies found high rates of hypertension among nonoverweight Asian adults (28,29). Clinicians should be aware that Asians may be at risk for hypertension and hypertension-related disease even at normal BMI. Furthermore, NHANES shows that Asian Americans have 1) lower levels of awareness of hypertension when their disease is diagnosed and 2) lower levels of adherence to hypertension medication than white or black Americans have (30). Because of the disproportionate share of death caused by cardiovascular and cerebrovascular disease among Asian American adults, screening and education are needed.

Strengths of our survey include its population-representativeness, objective measures of blood pressure, and the use of multiple languages in interviewing and examining participants to ensure inclusion of New York City’s diverse racial/ethnic minority populations. One limitation was the small sample size for some racial/ethnic subgroups, requiring us to combine certain subgroups (such as Mexicans, other Central Americans, and South American) to ensure reliability. Although the sampling design and statistical weighting process reduced the risk of selection bias, eligible participants who completed the study may have differed from those who did not. The distribution of unweighted demographic characteristics of our study participants was similar to census distributions (12). Finally, this study was cross-sectional, precluding any ability to infer cause-and-effect between characteristics of survey participants and prevalence of hypertension.

Our study underscores the need to disaggregate data for subgroups of Hispanic and Asian populations; overall population data may mask differences among subgroups. Targeted strategies for hypertension prevention and treatment are needed for various racial/ethnic subgroups, taking into account cultural practices, BMI-specific risks, and community awareness and support. Education for health care providers is also needed to raise awareness of subgroup differences and increase hypertension detection. The use of community health workers and the coordination of care can increase knowledge of cardiovascular disease and improve management of hypertension in racial/ethnic minority groups (25,31). Improved screening for hypertension, increased awareness of risk factors, and better hypertension management could mitigate the burden of hypertension on vulnerable racial/ethnic minority populations.

## Post-Test Information

To obtain credit, you should first read the journal article. After reading the article, you should be able to answer the following, related, multiple-choice questions. To complete the questions (with a minimum 75% passing score) and earn continuing medical education (CME) credit, please go to http://www.medscape.org/journal/pcd. Credit cannot be obtained for tests completed on paper, although you may use the worksheet below to keep a record of your answers.

You must be a registered user on http://www.medscape.org. If you are not registered on http://www.medscape.org, please click on the “Register” link on the right hand side of the website.

Only one answer is correct for each question. Once you successfully answer all post-test questions, you will be able to view and/or print your certificate. For questions regarding this activity, contact the accredited provider, CME@medscape.net. For technical assistance, contact CME@medscape.net. American Medical Association’s Physician’s Recognition Award (AMA PRA) credits are accepted in the US as evidence of participation in CME activities. For further information on this award, please go to https://www.ama-assn.org. The AMA has determined that physicians not licensed in the US who participate in this CME activity are eligible for *AMA PRA Category 1 Credits™*. Through agreements that the AMA has made with agencies in some countries, AMA PRA credit may be acceptable as evidence of participation in CME activities. If you are not licensed in the US, please complete the questions online, print the AMA PRA CME credit certificate, and present it to your national medical association for review.

## Post-Test Questions

### Study Title: Racial and Ethnic Subgroup Disparities in Hypertension Prevalence, New York City Health and Nutrition Examination Survey, 2013–2014

#### CME Questions

1. Your large medical group has decided to initiate care improvement programs for adults with hypertension, with a special focus on high-risk populations in your practice. What was the overall approximate prevalence of hypertension in the current study of adults in New York City?
  - 7%
  - 16%
  - 23%
  - 34%
2. Your practice treats a diverse patient population. Which of the following racial or ethnic groups had the ***highest*** age-standardized prevalence of hypertension in the current study?
  - Blacks
  - Whites
  - Asians
  - Hispanics
3. What did the multivariate logistic regression analysis reveal regarding racial and ethnic differences in hypertension in the current study?
  - Asians had the lowest odds of having hypertension
  - Hispanics had the highest odds of having hypertension
  - Puerto Ricans and Central/South Americans had particularly high odds for hypertension
  - Blacks and Asians had higher odds for hypertension compared with whites, but Hispanics did not
4. Along with hypertension, obesity is a major diagnosis in your practice. Overall, the prevalence of hypertension increased monotonically with body mass index in the current study among all groups ***except:***
  - Asians
  - Blacks
  - Whites
  - Hispanics
